# Two cases of spontaneous rupture of the uterine artery in the perinatal period: A case report

**DOI:** 10.1097/MD.0000000000033692

**Published:** 2023-05-17

**Authors:** Lingyun Hu, Jing Ning, Li’an Li, Yanping Lu, Yanqin You

**Affiliations:** a Department of Obstetrics & Gynecology, The First Medical Center of the PLA General Hospital, Beijing, China; b Department of Obstetrics & Gynecology, Sanya Maternal and Child Health Hospital, Sanya, Hainan, China.

**Keywords:** pregnancy, puerperium, spontaneous rupture, uterine artery

## Abstract

Uterine artery spontaneous rupture is a rare but potentially life-threatening complication during pregnancy and puerperium. The lack of typical symptoms makes it difficult to diagnose, which can result in serious consequences for both the mother and fetus.

**Patient concerns:**

Case 1 presented with fainting and lower abdominal discomfort, while Case 2 developed hypotension after delivery and remained in poor condition even after rehydration.

**Diagnoses:**

Both cases were diagnosed with uterine artery spontaneous rupture, with intraoperative findings revealing ruptures in different branches of the uterine artery.

**Interventions:**

Both cases underwent surgical interventions, with laparoscopic surgery performed in Case 1 and repair of the ruptured artery in Case 2.

**Outcomes:**

Both cases had successful outcomes, with the ruptured arteries repaired and the patients discharged from the hospital within a week after surgery.

**Lessons:**

Uterine artery spontaneous rupture is a rare but potentially life-threatening complication that may present with atypical symptoms. Early diagnosis and prompt surgical intervention are crucial in preventing serious complications for both the mother and fetus. Clinicians should maintain a high level of suspicion for this condition when evaluating patients presenting with unexplained symptoms or signs of peritoneal irritation during pregnancy and puerperium.

## 1. Introduction

Spontaneous ruptured bleeding from the uterine artery is rare and may lead to shock with serious implications. Most reported cases to occur in the third trimester of pregnancy. Also, the diagnosis of uterine rupture is usually made at the time of surgery. The typical symptoms of this condition are sudden onset of abdominal pain and signs of hypovolemic shock. However, no bleeding is shown, and a significant decrease in hemoglobin level is a common finding. Therefore, it is also important to identify which requires a combination of general surgery and gastroenterology doctors.

Therefore, to further clarify the condition of spontaneous uterine artery rupture and bleeding, we report 2 patients who presented with spontaneous uterine artery rupture in the perinatal period and study the etiology, site of bleeding, diagnostic modalities, and treatment options. This will provide further evidence for the diagnosis and treatment of this condition.

## 2. Case presentation

Case 1: The patient is 29-year-old, has had 2 pregnancies but never delivered, and has had 1 abortion. There was a history of mild dysmenorrhea and hypothyroidism for 5 years after treatment for hyperthyroidism, and she was taking eugenol. At 21 + 1 weeks of menopause, the patient felt a sensation of defecation at work and suddenly fainted after using the toilet, waking up half a minute later. Subsequently, the patient experienced lower abdominal discomfort with malaise, without nausea or vomiting. Subsequently, the patient presented to our emergency department. After admission, an ultrasound examination was performed, which showed a midterm pregnancy with a small amount of fluid in the pelvis. The patient’s hemoglobin was 97 g/L, and blood glucose was 5.4 mmol/L. Examination showed no abdominal pressure pain, rebound pain, or muscle tension. The fetal heart rate was also 145 beats per minute. The general surgery consultation did not rule out the possibility of gastroparesis. Because the cause of the syncope was unknown, the patient was admitted to the observation room, and intravenous access was established for rehydration therapy. Seven hours later, the patient reexperienced syncope after a bathroom break while continuous electrocardiogram monitoring was performed, and a catheter was immediately placed. The patient reported abdominal distention, which was more pronounced than before, as well as significant abdominal pressure, rebound pain, and muscle tension. The patient had an indistinct fundus on palpation, little uterine tone, a fetal heart rate of 140 to 166 beats/minutes, and palpable irregular uterine contractions of moderate to weak intensity. Repeat blood routine, coagulation function, biochemical index, blood amylase, and bedside ultrasonography showed no abnormalities in the liver, gallbladder, pancreas, spleen, kidney, or ureter. The patient’s peritoneal effusion was significantly increased. The depth of the left abdomen was approximately 1.6 cm, the right abdomen was approximately 2.7 cm, and there appeared to be a floating mass in the left lower abdomen. A laparotomy was performed on the patient, and 5 mL of non-coagulated blood was punctured. A green channel for maternal resuscitation was initiated, and immediate preparations were made for exploratory laparoscopic surgery under general anesthesia. Before surgery, the patient’s hemoglobin was 87 g/L. During surgery, approximately 3800 mL of blood and clots were found. The uterus was pale in color, and the left parametrial bleeding was active after the clot aspiration. However, due to the enlarged uterus during pregnancy, exposing the bleeding site and the bleeding was difficult. During the procedure, laparoscopic surgery was converted to open surgery. The patient could be seen to have a ruptured ascending branch of the left uterine artery with visible adhesions around it and a rougher peritoneum next to the adhesions. The patient was ligated 3 times with vascular ligature forceps, and the ascending branch of the left uterine artery was reinforced with continuous sutures using 2 to 0 absorbable sutures. The fetal heart rate was 50 to 53 beats/minutes during the operation, and the hemoglobin was 83 g/L. Postoperatively, the patient was given ventilator-assisted breathing, and the indwelling catheter, abdominal drainage tube, and tracheal intubation were transferred to the intensive care unit. The fetal heartbeat was 142 beats per minute, and contractions could be palpated. Postoperatively, the patient received 600 mL of plasma, 900 mL of suspended red blood cells, and 200 mL of platelets. On the second postoperative day, the tracheal intubation was removed. She was transferred back to the general obstetric ward on the 4th postoperative day. The sutures were removed and discharged on the 7th postoperative day. Due to the patient’s heavy bleeding and intraoperative fetal heart rate of 50 to 53 beats per minute, the pregnant woman and her family requested termination of the pregnancy. Subsequently, amniotic fluid injection was performed to induce labor at 26 weeks of gestation.

Case 2: The patient was 38 years old and had 3 pregnancies and 1 delivery. At 39 + 4 weeks of menopause, the patient presented with a bloody vaginal discharge and irregular abdominal pain for 1 day and was admitted to the hospital. Physical examination showed a uterine height of 33 cm, an abdominal circumference of 97 cm, a fetal heart rate of 142 beats/minutes, and a medium, soft, 1.5 cm long cervix. The opening of the cervix could accommodate 1 finger, the head was first exposed, and the fetal heart rate was monitored for NST response. The patient was spontaneously delivered at 39 + 5 weeks s of pregnancy at 6:00 AM. The newborn was transferred to the NICU. The patient’s postpartum blood pressure was 89/63 mm Hg, and her heart rate was 87 beats/minutes.

Thirty minutes after delivery, the patient felt fatigued and had dark eyes. The patient’s blood pressure was 80/53 mm Hg, and her heart rate was 112 beats/minutes. Physical examination revealed a soft abdomen with no pressure pain, rebound pain, or myalgias, subungual fingers at the base of the eyes, good contractions, and little vaginal bleeding. The patient was given an IV, continuous electrocardiogram monitoring, and an indwelling urinary catheter. A little more than 1 hour after delivery, the woman felt increased fatigue, her blood pressure was 66/35 mm Hg, and her heart rate was 112 beats/minutes. After rapid rehydration therapy, blood pressure was 95/60 mm Hg, and heart rate was 79 beats/minutes. Physical examination revealed a pale, indifferent, conscious patient with abdominal distension, no pressure pain, rebound pain, and muscle tension. The patient’s hemoglobin was 81 g/L. The patient was given oxygen by face mask and injected with norepinephrine. Bedside ultrasound showed free fluid in the hepatorenal recess, perihepatic recess, splenorenal recess, and pelvis, and ascites were approximately 6.0 cm deep. Five mL of non-coagulated blood was withdrawn by laparotomy, followed by the resuscitation of q his. Immediate laparoscopic surgery was performed under general anesthesia.

**Figure 1.**
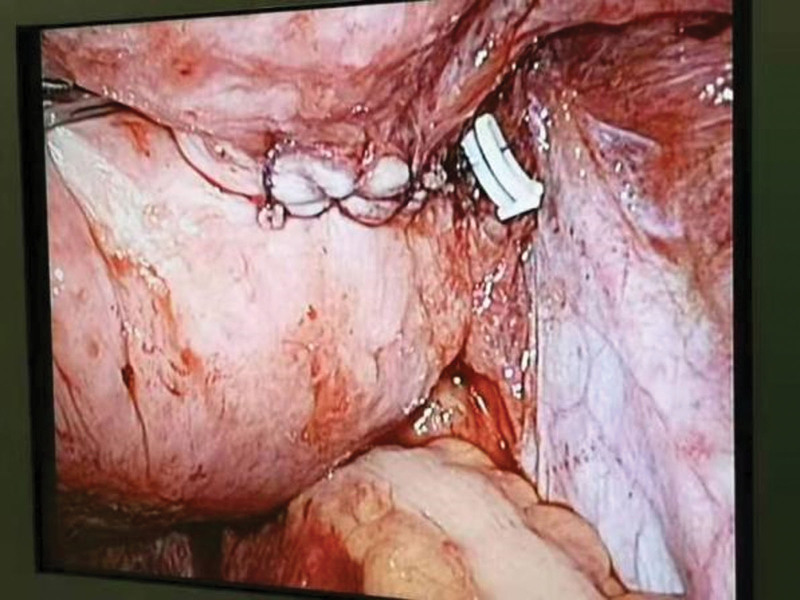
Suture after rupture of descending branch of right uterine artery.

During the procedure, a large amount of blood (about 4000 mL) was seen in the pelvic and abdominal cavities. After aspiration of the dark red blood from the pelvic and abdominal cavities, the right wall of the lower uterine segment was seen to become brittle and deteriorated. Active bleeding was seen in the descending branch of the right uterine artery. The patient was serially sutured with 2 to 0 absorbable sutures to rupture the vessel, and the vessel was ligated with forceps and tied once (Fig. 1). The intraoperative emergency hemoglobin was 67 g/L. The patient received 2000 mL of crystalloid fluid, 500 mL of colloid fluid, 570 mL of plasma, and 900 mL of suspended red blood cells. Postoperatively, the patient was transferred to the intensive care unit. The tracheal intubation was removed on the second postoperative day, and the patient was returned to the general obstetric ward. The patient was discharged on postoperative day 7.

## 3. Discussion

Spontaneous rupture of uterine vessels in the perinatal period is rare. The typical clinical manifestations of patients are sudden abdominal pain, hypovolemic shock, and decreased hemoglobin.^[1]^ In this paper, case 2 mainly manifests as hypovolemic shock and decreased hemoglobin, while case 1 has no obvious hypovolemic shock and decreased hemoglobin. We consider that it may have a certain oppressive effect on the enlarged pregnant uterus and pelvic wall, so the decrease of hemoglobin in case 1 is not fast, and the changes in heart rate and blood pressure are not very obvious. However, after paracentesis, the patient was found to have intra-abdominal hemorrhage and was immediately transferred from the emergency department to the operating room. During the transfer process, the pregnant uterus has a certain activity, resulting in a certain amount of bleeding. This was confirmed during laparoscopic exploration. After finding the suspicious bleeding site, there was obvious active bleeding when the uterus was pushed to the right to expose the left parametrium. However, there was no obvious active bleeding after restoring the original anatomical structure. Therefore, there is a difference between the estimated postoperative and intra-abdominal bleeding volumes in the emergency department. However, both cases still showed hypovolemic shock and decreased hemoglobin.

Moreover, the disease is rare, and the enlarged uterus causes blood to flow to the middle and upper abdomen, so it is difficult to diagnose before surgery. Therefore, paracentesis should be performed in time in the event of sudden abdominal pain, abdominal distension, hypovolemic shock, decreased hemoglobin, or peritoneal irritation and moving dullness during pregnancy. Once the non-coagulated blood is drawn and the related differential diagnosis diseases are excluded, the possibility of the disease should be highly vigilant.

The etiology of the spontaneous rupture of uterine vessels in the perinatal period is unknown. According to reports in the literature, it may be related to hemodynamics,^[1]^ anatomical factors,^[2]^ and hormones.^[3]^ During pregnancy, uterine blood supply increases, and venous blood flow is slow, compressing the inferior vena cava, blocking pelvic blood flow, and increasing uterine venous pressure. More importantly, the uterine serosal surface veins are superficial, with thin walls, lacking a shell composed of fascia, and no venous valves. Therefore, perinatal uterine blood vessels are prone to rupture and hemorrhage. In domestic and foreign literature reports, it is manifested in variceal bleeding at the uterine horn during pregnancy. In addition, according to reports in foreign literature, when there was endometriosis or pelvic inflammation in the past, the subserous vein or parametrial blood vessels of the uterus were more superficial, tortuous, and distended or even exposed. Therefore, it is also one of the high-risk factors for the spontaneous rupture of uterine arteries in the perinatal period. Endometriosis is a chronic inflammatory disease that often affects the peritoneum, ovaries, and rectovaginal septum but can also affect the abdomen or other sites outside the abdomen. Endometriosis or pelvic inflammation-related complications in the third trimester are rarely reported. A review of a few cases report it suggests that spontaneous vascular rupture is the most serious complication.^[4–7]^ According to literature reports, 61% of spontaneous ruptures of the uterine arteries occur during pregnancy, 18% during labor, and 21% after delivery.^[8–10]^

Case 1 was a ruptured uterine artery during pregnancy, and the patient suddenly developed syncope after a toilet visit. Intraoperative exploration showed that the left parametrium formed membranous adhesions with the left pelvic wall, and the left uterine artery was tortuous and exposed at the parametrium. A rough surface of about 2*3 cm can be seen on the left basin wall, shaped like transparent blisters. Because the patient had a history of dysmenorrhea and the left pelvic wall was seen during the operation, it was considered that the patient had early endometriosis manifestations. Consequently, the exposed, tortuous uterine artery is avulsed after the patient changes position, leading to bleeding. In case 2, the patient experienced fetal heart rate deceleration during the second stage of labor and did not recover. The patient’s position was changed to thigh flexion after fetal suction-assisted delivery. However, there was no postpartum change in the patient’s position. Therefore, it is considered that the bleeding time of case 2 may have occurred during labor. In this case, the right-side wall of the lower uterine segment was seen to be brittle on the posterior surface during the operation, and it seemed to be avulsed from the lateral pelvic wall. Combined with the patient’s history of dysmenorrhea, there was a history of childbirth and induced abortion. Therefore, the possibility of pelvic inflammatory disease cannot be ruled out. Both cases had features of pelvic inflammation, which were considered to be related to the causes mentioned above.

In case 1, a small amount of peritoneal effusion was indicated by ultrasonography when he presented to the emergency department. Moreover, the patient’s blood pressure was 98/65 mm Hg, the heart rate was 72 beats/minutes, and the hemoglobin was 97 g/L, but hemoperitoneum was not considered. If multidisciplinary consultation is carried out at this time, an abdominal puncture should be performed as soon as possible, the diagnosis time should be clarified, and the transfer process should be carried out as smoothly as possible to reduce patients’ blood loss effectively. The second case occurred after the multiparous woman gave birth. Although it is fetal suction midwifery, due to the rarity of the disease, abdominal hemorrhage was not considered after the blood pressure drop. It did not attract enough attention in the early stage of the disease, thus delaying it. Therefore, for pregnant women with a history of multiple abdominal operations, miscarriage, delivery, and endometriosis, such as sudden abdominal pain and symptoms of hypovolemic shock during pregnancy, childbirth, or postpartum, effective measures should be taken as soon as possible. Means. In conclusion, early diagnosis, surgery, and bleeding control are the only effective measures to improve maternal and infant outcomes.

## Author contributions

**Conceptualization:** Lingyun Hu, Jing Ning, Yanqin You.

**Data curation:** Lingyun Hu, Jing Ning, Yanqin You.

**Formal analysis:** Jing Ning, Li’an Li.

**Funding acquisition:** Li’an Li.

**Investigation:** Yanping Lu.

**Methodology:** Yanping Lu.

**Project administration:** Yanping Lu.
